# Biological and Physical Performance Markers for Early Detection of Cognitive Impairment in Older Adults

**DOI:** 10.3390/jcm13030806

**Published:** 2024-01-30

**Authors:** Hanna Kerminen, Emanuele Marzetti, Emanuela D’Angelo

**Affiliations:** 1 Faculty of Medicine and Health Technology, Gerontology Research Center (GEREC), Tampere University, Arvo Ylpön katu 34, 33520 Tampere, Finland; hanna.kerminen@tuni.fi; 2 Department of Geriatrics, Orthopedics and Rheumatology, Università Cattolica del Sacro Cuore, L.go F. Vito 1, 00168 Rome, Italy; 3 Fondazione Policlinico Universitario “Agostino Gemelli” IRCCS, L.go A. Gemelli 8, 00168 Rome, Italy; emanuela.dangelo@policlinicogemelli.it

**Keywords:** aging, chronic inflammation, cognitive frailty, dual task, gait, geroscience, inflammation, motoric cognitive risk syndrome, neurodegeneration, neurofilament

## Abstract

Dementia is a major cause of poor quality of life, disability, and mortality in old age. According to the geroscience paradigm, the mechanisms that drive the aging process are also involved in the pathogenesis of chronic degenerative diseases, including dementia. The dissection of such mechanisms is therefore instrumental in providing biological targets for interventions and new sources for biomarkers. Within the geroscience paradigm, several biomarkers have been discovered that can be measured in blood and that allow early identification of individuals at risk of cognitive impairment. Examples of such markers include inflammatory biomolecules, markers of neuroaxonal damage, extracellular vesicles, and DNA methylation. Furthermore, gait speed, measured at a usual and fast pace and as part of a dual task, has been shown to detect individuals at risk of future dementia. Here, we provide an overview of available biomarkers that may be used to gauge the risk of cognitive impairment in apparently healthy older adults. Further research should establish which combination of biomarkers possesses the highest predictive accuracy toward incident dementia. The implementation of currently available markers may allow the identification of a large share of at-risk individuals in whom preventive interventions should be implemented to maintain or increase cognitive reserves, thereby reducing the risk of progression to dementia.

## 1. Introduction

The aging of the population is an emerging phenomenon in contemporary societies. This demographic transition challenges the sustainability of health and social care systems that are largely unprepared to deal with the medical needs of clinically complex older adults [1]. Indeed, disease-based healthcare services are unsuitable for comprehensively addressing the requirements of patients with multiple diseases, geriatric syndromes, and functional/cognitive decline [2]. These individuals would instead benefit from a personalized care approach that allows the consideration of all factors that influence their health and well-being. To deliver optimal medical care to these “modern” patients, a deeper comprehension of the mechanisms underlying the aging process and the devising of interventions that modify their trajectories are of the utmost importance. Indeed, the biological pathways that drive the aging process are now recognized as key factors underpinning the pathogenesis of most chronic degenerative diseases [3]. The aging process has a unique course across individuals which leads to a remarkable heterogeneity in biological age among persons of the same chronological age [4,5]. As a result, some older adults remain relatively healthy and functionally independent until an advanced age [6], while others experience multimorbidity and functional impairment at the early retirement age [7,8]. Notwithstanding, only a minority of older adults manage to show no disability until the end of their lives [9].

Disabling conditions cause significant psychosocial consequences in older adults and their families as well as economic and resource-related burdens for societies [10,11]. Cognitive disorders and dementia are the seventh leading cause of death globally and among the most important determinants of functional impairment and disability in older adults [12,13]. Because aging is a major risk factor for different types of cognitive decline and dementia, the number of people suffering from cognitive impairment is increasing rapidly due to population aging [14]. Therefore, the prevention of cognitive decline and dementia has become a global public health priority [12,13,15]. The elimination or management of modifiable risk factors for dementia (e.g., obesity, smoking, excessive alcohol consumption, hypertension, diabetes, depression, social isolation, physical inactivity) and the maintenance of cognitive reserve capacity are accessible methods for preventing cognitive impairment [16]. However, there is an urgent need for novel preventive and therapeutic strategies, including disease-modifying drugs [17].

In the last two decades, there has been an increasing interest in geroscience, a biomedical research field that attempts to understand how the aging process leads to chronic diseases in order to devise interventions that prolong the lifespan and delay the onset of diseases as people age [18,19]. According to the geroscience paradigm, therapies that target fundamental aging mechanisms, such as cellular senescence, have the potential to postpone the development of chronic illnesses and thereby extend the healthspan [20]. One focus of geroscience is to clarify the biological mechanisms of aging that are linked to cognitive impairment. Senolytic drugs that act by eliminating senescent cells have been shown to promote healthy aging and halt the progression of Alzheimer’s disease (AD) in animal models [21]. Several senolytics (e.g., dasatinib + quercetin) are currently being tested for safety and efficacy in clinical trials. Additional investigational drugs with disease-modifying potential are those targeting neuroinflammation, oxidative stress, and neuroplasticity [22].

As of now, however, there are no curative treatments available for fully developed cognitive disorders. Yet, potentially reversible predementia syndromes exist that are associated with an increased risk of progression to dementia. A challenge of detecting cognitive disorders at their early stages is related to the fact that pathological changes of the nervous system develop slowly during years or even decades, and clinically measurable cognitive symptoms appear not until the chronic phase of the disease. The optimal situation would allow the identification of emergent cognitive disorders at their reversible stages when the development of cognitive impairment could still be prevented. In addition, there might be opportunities to intervene in the underlying pathological processes to prevent the development of dementia. To accomplish these objectives, there are still challenges to overcome. First, reliable biomarkers are needed to detect cognitive disorders at their very early stages. Second, it is necessary to discover interventions that have preventive impact on disease processes and progression.

Accumulating evidence indicates that both biological markers of aging, especially those related to chronic inflammation, and measures of physical performance may allow the early detection of individuals at increased risk of developing cognitive disorders. Because the same functional brain areas, the frontal and prefrontal lobe-related brain networks, are responsible for gait control and cognitive functions, gait changes often accompany cognitive decline, even so that gait abnormalities are detectable long before the onset of measurable cognitive signs or symptoms [23]. This narrative review aims to discuss the current knowledge of biomarkers for the early detection of emergent cognitive disorders. We will focus on biological markers of aging associated with predementia states and on those of physical performance that may be used to predict the risk of cognitive impairment.

## 2. Biological Aging and Its Relationship with Physical and Cognitive Frailty

### 2.1. Hallmarks of Aging and Their Interaction with Life Course Determinants

Twelve hallmarks of aging have been proposed to describe the molecular and cellular mechanisms of biological aging [3]. The primary hallmarks reflect irreversible cellular damage that accumulates with time to the genome, telomeres, epigenome, proteome, and cellular organelles. These hallmarks include genomic instability, telomere attrition, epigenetic alterations, loss of proteostasis, and defective macroautophagy. The antagonistic hallmarks are related to cellular responses to these accumulated damages and encompass deregulated nutrient sensing, mitochondrial dysfunction, and cellular senescence. Finally, integrative hallmarks are the result of the uncompensated effects of primary and antagonistic hallmarks and include stem cell exhaustion, altered intercellular communication, chronic inflammation, and dysbiosis.

**Figure 1:**
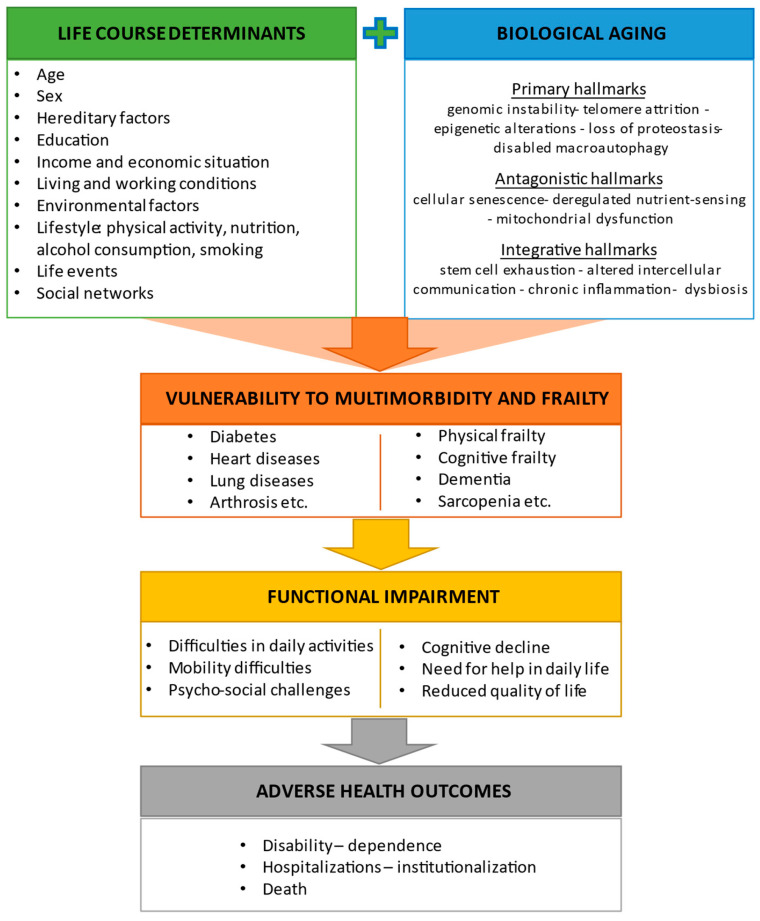
The interplay between life-course determinants and mechanisms of biological aging makes older adults vulnerable to chronic diseases, frailty, and negative health-related outcomes.

In addition to biological alterations, advancing age involves significant changes in social roles and positions [24] as well as psychological adaptations that are necessary to cope with physiological and socioeconomic modifications that occur over the life course [25]. The interplay between biological, genetic, physical, social, psychological, and environmental factors affects the process of aging and increases the vulnerability of older adults to chronic diseases, functional impairment, and negative health-related outcomes [26] (Figure 1).

Environmental factors that impact the aging process and the development of chronic diseases comprise natural, built, and social environments as well as lifestyle factors [27]. At the same time, the interconnection between the biological mechanisms of aging and environmental factors offers several opportunities to intervene in the process of aging and the pathogenesis of chronic diseases. Indeed, recent research suggests that chronic inflammation is a major mediating factor in the pathogenesis of chronic diseases induced by environmental factors [28]. Multicomponent interventions including nutritional therapy, physical exercise, and psychosocial support are effective for preventing chronic diseases [29], and their effect may be, at least partly, related to their ability to reduce chronic inflammation [30].

### 2.2. Frailty, Cognitive Frailty, and Other Predementia Syndromes

Frailty is prevalent among older adults and is related to negative health-related events, such as functional impairment, disability, hospitalizations, institutionalization, and mortality [31]. Frailty is a multifactorial and complex condition in which an individual’s ability to resist stressful events is reduced due to cumulative age-related declines in multiple physiological systems [32,33]. Unlike “normal aging”, which is characterized by a gradual decrease in physiological reserve capacities across organ systems, the rate of decline in organ functions is accelerated in frailty. As a result, older adults living with frailty are exposed to disproportionate changes in their health and functional status even when challenged by minor stressors [32]. Frailty is potentially reversible at least in its early stages [34,35] and, therefore, should be detected and managed in a timely manner.

A single operational definition of frailty is still unavailable owing to different perspectives on its conceptualization. The most widely used paradigms are the phenotypic model by Fried et al. [36] and the cumulative deficit model by Rockwood et al. [37]. In the phenotypic model, frailty is identified based on five predetermined physical factors: unintentional weight loss, weakness, slowness, self-reported exhaustion, and low levels of activity. Of these five factors, having one or two defines a condition of prefrailty, while the presence of three or more is indicative of frailty [36]. In the cumulative deficit model, frailty is defined as the cumulative effect of health deficits. The more health deficits an individual accumulates, the frailer the person is [37].

Frailty and cognitive impairment share similar biological pathways and are often interconnected [38]. Therefore, in an attempt to prevent cognitive impairment, it is necessary to consider not only cognitive resources but also the physical domain of an older individual. Indeed, there are a few potentially preventable predementia syndromes in which physical performance deterioration co-occurs with subtle or mild cognitive changes, such as cognitive frailty, motoric cognitive risk syndrome (MCR), and physio-cognitive decline syndrome (PCDS) [39,40,41,42,43,44]. Cognitive frailty is characterized by the simultaneous presence of physical frailty and mild cognitive impairment (MCI) that does not fulfil the diagnostic criteria for dementia [39]. MCR is a clinical condition that encompasses slowness of gait and subjective cognitive complaints in the absence of cognitive impairment or disability [45]. PCDS is a recently described condition with concurrent cognitive impairment in any domain (≥1.5 standard deviation below age-, sex-, and education-matched norms) and slow gait or/and weak handgrip strength without mobility disability [43]. Thus, as indicated previously, an assessment of physical performance may assist in the early detection of cognitive decline. The relevance of measures of physical performance to the early identification of cognitive impairment is further discussed in a dedicated article section.

## 3. Biomarkers of Aging Associated with Cognitive Frailty or Cognitive Decline

### 3.1. Inflammatory Markers

Evidence indicates that chronic inflammation is associated with an increased risk of cognitive decline and dementia in older adults [46,47]. Although inflammation is a necessary defense mechanism against insults such as traumas, tissue injury, and external pathogens, a chronic inflammatory status may predispose a person to cognitive decline. In this context, it becomes a priority to understand whether age-related inflammatory markers mediate the relationship between certain risk factors and cognitive outcomes. The original studies of aging biomarkers related to cognitive frailty or cognitive decline are summarized in Table 1.

Systemic levels of interleukin (IL)-6 and C-reactive protein (CRP), two inflammatory markers extensively explored in epidemiological studies on aging [68], were associated with physical and cognitive decline in a representative sample of 415 community-dwelling older adults enrolled in the BELFRAIL study [49]. The association was found to be stronger for IL-6 than for CRP. In a recent meta-analysis, Groeger et al. [52] examined the association of circulating IL-6 and CRP levels with MCR. The results showed that higher concentrations of IL-6 and CRP were associated with an increased risk of MCR. A longitudinal study conducted by Bai et al. [48] explored the association between CRP and MCR subtypes (defined by the presence or absence of memory impairment) in community-dwelling older adults. Higher CRP levels were associated with MCR with memory impairment but not with MCR without memory impairment.

The underlying biological mechanisms of MCR are not yet fully understood. Research has shown that elevated levels of inflammatory markers are associated with an increased risk of developing the main components of MCR. Therefore, it is conceivable that genetic variants in the neuroinflammatory pathway may increase the risk of developing MCR. This possibility was explored by Sathyan et al. [55] in an investigation involving 530 individuals 65 years or older without MCR or dementia at baseline enrolled in the LonGenity study. Over a median follow-up of three years, 70 (13.2%) participants developed MCR. Of the 62 genetic variants in the neuroinflammatory pathway that were explored, single-nucleotide polymorphisms in the transcriptional regulatory region of the *IL-10* gene were associated with greater risk of incident MCR. This finding points to a possibile role of *IL-10* in the pathogenesis of dementia through the MCR pathway.

In another study, Merchant et al. [54] explored the association of MCR with body composition abnormalities, including sarcopenia, and systemic inflammation. The results showed that plasma levels of growth differentiation factor 15 (GDF15) and tumor necrosis factor alpha (TNF-α) were high and progranulin/TNF-α and IL-10/TNF-α ratios were low in MCR. TNF-α was significantly elevated in MCR independent of sarcopenia but without obesity, while low IL-10 levels and IL-10/TNF-α ratio were associated with MCR, independent of sarcopenia and body composition. In the European study to establish bioMARKers of human AGEing (MARK-AGE), the relationship between plasma GDF15 levels, cognitive frailty, and depression was explored in 2736 participants, including individuals who were 55+ years and younger adults [53]. High circulating levels of GDF15 were significantly and independently associated with a greater risk of both cognitive frailty and depression in the whole sample as well as in older adults. Several other studies demonstrated an association between depression and MCR [69,70,71] as well as between depression and dementia [72,73]. The link between depression and MCR could be mediated, at least partly, by inflammatory cytokines, changes in body composition, and reduced muscle strength [70]. As discussed later, the evaluation of these parameters may assist in the early indentification of individuals at risk of cognitive impairment.

### 3.2. Markers of Neurodegeneration

Neurofilament proteins (NfPs) are cytoskeletal components of neurons that are highly expressed in axons. Low amounts of NfPs are continuously released from neurons under physiological conditions, but their levels in both cerebrospinal fluid (CSF) and blood rise substantially in response to neuroaxonal injury independent of the underlying cause [74]. The neurofilament light chain (NfL) is the most extensively studied subtype of neurofilaments and the most soluble and abundant in the CSF and blood [75,76]. NfL levels increase linearly with age and may serve as a valid biomarker of neurodegeneration. Recent studies have demonstrated that circulating NfL levels can be used to discriminate between different preclinical stages of cognitive decline and to predict incident cognitive decline (Table 1). Furthermore, higher levels of NfL in blood or CSF have been associated with lower scores on the Mini Mental State Examination (MMSE) and worse executive function [77,78]. Higher plasma NfL concentrations are also associated with an increased risk of developing dementia [57]. These findings indicate that NfL dysregulation might contribute to the development of dementia and that its CSF and circulating levels could allow identification of preclinical stages. Furthermore, measures of plasma NfL levels could potentially be used to monitor the progression of cognitive decline.

### 3.3. “Next-Generation” Biomarkers

The interest in extracellular vesicles (EVs) and their cargos as well as in epigenetic markers has risen substantially in recent years owing to the recognition of their potential as biomarkers of aging and neurodegeneration. EVs are mediators of cellular communication and comprise a heterogeneous group of vesicles that are released by almost all cell types and are present in most body fluids. EVs are associated with both physiological and pathological conditions [79] through the transfer of their cargo, including proteins, lipids, and nucleic acids (e.g., DNA fragments, microRNA transcripts, noncoding RNAs, long noncoding RNAs, microRNAs (miRNAs), circular RNAs) to target cells [80,81]. Visconte et al. [58] recently showed that the plasma concentration of microglial-derived EVs was increased in individuals with cognitive frailty compared with healthy controls. These EVs were found to have a remarkable neurotoxic effect on neurons (Table 1). These findings suggest that an altered release of microglial-derived EVs may play a role in the pathogenesis of cognitive frailty and might therefore serve as biomarkers for early identification of individuals at risk of cognitive impairment.

miRNAs are noncoding RNAs that play important roles in regulating gene expression in most physiological processes as well as in pathways relevant to aging, such as cellular senescence [82]. There is preliminary evidence that some miRNAs (e.g., miRNA-206) could be used as molecular signatures of AD progression in individuals with MCI (Table 1) [59,60,61,62]. Further research is needed to clarify the ability of miRNAs to predict cognitive impairment in other predementia states.

The term “epigenetics” refers to chemical changes and configurations of the genome that impact its function. Studies have shown that the analysis of DNA methylation status at specific sites allows biological age to be accurately estimated [83]. Algorithms based on the degree of methylation at specific points of the genome have been developed to capture information on biological aging. These methylation algorithms are divided into three generations of clocks. The first generation of methylation algorithms aims to estimate chronological age [84]. Second-generation algorithms pursue the prediction of age-related phenotypes and mortality [85]. Finally, the third-generation clocks are built to compute the rate of biological aging. Several studies have examined the association of DNA methylation algorithms with cognitive impairment and dementia (Table 1). Sugden et al. [63] showed that the association of a third-generation epigenetic clock (DunedinPACE) with cognitive decline was stronger than for first- and second-generation clocks. Adults with a diagnosis of dementia or MCI had a faster DunedinPACE compared with those without cognitive impairment. What is more, a faster DunedinPACE in midlife was associated with greater risk of cognitive decline in old age. Degerman et al. [64] found that a younger epigenetic age was associated with better episodic memory and might protect againt cognitive loss in advanced age. Altogether, available evidence indicates that third-generation blood-based DNA methylation measures could be used to gauge the risk of cognitive decline.

### 3.4. Proteomic Biomarkers

Several circulating proteins are useful biomarkers for calculating the rate of aging and identifying individuals at greater risk of chronic diseases, including dementia (Table 1). Using data from the Invecchiare in Chianti (InCHIANTI) study, Tanaka et al. [65] identified four plasma proteins (i.e., peptidase inhibitor 3 (PI3), trefoil factor 3 (TFF3), pregnancy-associated plasma protein A (PAPPA), and agouti-related peptide) that were associated with increased odds of cognitive impairment or dementia at baseline. PI3 showed the strongest association with cognitive impairment and dementia, possibly due to its role in the regulation of inflammation. Two additional proteins (myostatin and integrin aVb5) were instead related to lower chances of baseline cognitive impairment or dementia. Interestingly, plasma concentrations of myostatin, PI3, TFF3, and PAPPA were found to be associated with cognitive decline in participants who were cognitively healthy at baseline. Findings were validated in two independent cohorts, the Baltimore Longitudinal Study of Aging (BLSA) and the Religious Orders Study (ROS).

In a subsample of 236 participants of the Alzheimer’s Disease Neuroimaging Initiative (ADNI), DeMarshall et al. [66] found that a panel of serum autoantibodies measured by protein microarray could distinguish individuals with AD-associated MCI from age- and sex-matched controls with an accuracy of 100%. The authors also showed that the autoantibody panel was highly specific for MCI, such that it distinguished between participants with AD-associated MCI and those with other neurodegenerative diseases (e.g., Parkinson’s disease, multiple sclerosis) and non-neurodegenerative conditions (e.g., early-stage breast cancer). In a recent study, Ehtewish et al. [67] explored the plasma autoimmune profile of 127 older adults of whom 50 were cognitively healthy, 55 had MCI, and 22 had a diagnosis of dementia. Differential expression analysis revealed 33 dysregulated plasma autoantibodies in participants with dementia compared with cognitively healthy controls, while 38 dysregulated autoantibodies differentiated individuals with dementia from those with MCI. Five autoantibodies (i.e., anti-CAMK2A, CKS1B, ETS2, MAP4, and NUDT2) were found to be dysregulated in both dementia and MCI.

These encouraging findings call for future studies aimed at establishing whether proteomic biomarkers may be used in clinical settings to identify individuals at risk of cognitive impairment.

## 4. Frailty and Physical Performance as Predictors of Cognitive Decline

### 4.1. Frailty

Several longitudinal studies have shown that older adults living with physical frailty have an increased risk for incident cognitive disorders [86,87,88,89], even independent of brain atrophy or cerebral small vessel disease on magnetic resonance imaging (MRI) [87] (Table 2). Older adults with physical frailty who experience subjective cognitive decline (SCD) without objective cognitive impairment are at increased risk of developing cognitive disorders, in particular vascular dementia [88]. An especially high risk for dementia is associated with cognitive frailty, i.e., simultaneous physical frailty and MCI [89]. These observations imply that a common geriatric metric such as frailty may be used by clinicians to decide on further testing to uncover an initial decline of cognition as well as to devise preventive strategies and plan follow-up assessments.

### 4.2. Gait Speed, Stride-to-Stride Variability, and Gait Speed under Dual-Task Conditions

Walking is a complex task that requires the coordination and integration of functions across multiple organ systems including the nervous, sensory, musculoskeletal, and cardiorespiratory systems [23]. Alterations or deficits in one or more of these interrelated systems may lead to gait changes and abnormalities. Although moderate gait changes are part of normal aging, older adults are vulnerable to pathological gait abnormalities from multifactorial reasons [105]. Notably, gait changes in older age are associated with several adverse consequences, such as cognitive disorders, mobility disability, hospitalizations, institutionalization, and death [23,106,107].

In a recent systematic review and meta-analysis, Andrews et al. [108] examined the comfortable gait speed in community-dwelling healthy adults. The mean comfortable gait speed in young adults was 1.38 m/s in women and 1.40 m/s in men and it slowed through the adult years. In those older than 80 years, the mean gait speed was 0.97 m/s in women and 1.05 m/s in men. Hence, older adults tend to walk slower than before even if they were capable of walking fast. A possible reason for the slowing of comfortable gait speed is related to the fact that walking fast requires increased activation of motor regions at the frontal cortex in older adults compared with younger adults [109]. Slower walking is also related to a cautious gait that is typical of older adults. A cautious gait ensures balance by increasing step width and reducing gait speed, stride length, and cadence [110].

Consistent evidence from cross-sectional studies suggests that there is an association between a slow comfortable gait speed and declining cognitive function [95,111,112,113,114,115]. Studies have shown that a person’s self-selected fast-paced gait speed correlates better with cognitive decline than usual, comfortable gait speed [93,116,117]. The reason for that might be the diminished cognitive resources in individuals with cognitive decline, as walking fast is a resource-demanding task.

Another commonly used measure is gait variability, i.e., the stride-to-stride fluctuations in walking. Results regarding changes in gait variability with age differ across studies. Some researchers have found that gait variability increases with age [118], but several other studies have shown that intraindividual gait variability remains stable over adulthood [110]. Cross-sectional studies confirmed an association between high gait variability and lower cognitive function [114,115,119,120,121,122,123]. In particular, a high gait variability was associated with AD and Lewy body dementia [114,124,125]. In addition, a high gait variability and poor cognition are associated with thinner cortical gray matter on brain MRI [120]. Similarly, a decreased gray matter volume of the right hippocampus on brain MRI was associated with both slowing of gait and cognitive impairment [97].

Although a slowing of gait speed in old age is a physiological phenomenon, the effect of pathological processes on comfortable gait speed is remarkable. Generally, a high gait variability is associated with slow gait speed [126]. Obviously, gait slowing has a multifactorial background, but cognitive decline, even if subclinical, tends to slow down gait speed and increase intraindividual gait variability.

Various longitudinal studies have examined the predictive ability of gait abnormalities toward incident cognitive frailty or cognitive impairment (Table 2). Verghese et al. [91] found that neurologic gait abnormalities (i.e., unsteady gait, frontal gait, hemiparetic gait, neuropathic gait, ataxic gait, parkinsonian gait, and spastic gait) predicted incident vascular dementia over 6.6 years of follow-up. There is strong evidence showing that a slower gait speed is associated with the later development of cognitive decline or dementia [92,94,95,96,98,100]. Several longitudinal studies found that simultaneous slow gait speed and subjective cognitive complaints predict the development of cognitive impairment and dementia [40,45,104,119]. In addition, Rosso et al. [97] found that a gradual decline in gait speed over a 14-year follow-up predicted incident MCI or dementia in initially healthy older adults. More recently, Hwang et al. [100] demonstrated that slower gait speed, lower balance confidence, and greater double-support time during walking predicted incident cognitive frailty over a two-year follow-up. Furthermore, a greater gait variability was linked with incident MCI during four years of follow-up [101].

The dual-task paradigm is based on the observation that the initiation of a second task during a motor or cognitive activity leads to a decreased performance in one or both tasks due to competition between the attentional resources available. In healthy older adults, both cognitive and motor performances decline during a dual-task walk compared with simply walking [127]. However, research has shown that higher dual-task costs, i.e., the magnitude of deterioration in gait performance measured during dual-task walking compared to single-task walking, are typically seen in those with cognitive impairment (Table 2) [23,102,128,129,130]. Indeed, intact executive functions, i.e., higher cognitive functions that are used to allocate attention between different tasks, are necessary to perform simultaneous motoric and cognitive tasks successfully [129]. Performing a cognitive task during walking may overload the attentional resources available, leading to disruptions in both cognitive performance and gait in individuals with even minor or subclinical deficits in executive functions. For this reason, several prospective studies have shown that a dual-task walk is superior to a single-task walk in detecting an incipient cognitive decline [102,129,130]. When cognitive impairment is more severe, there might be no added value in a dual-task walk compared with single-task walk [115,123,131].

In the Gait and Brain Study, Montero-Odasso et al. [102] explored gait parameters predictive of dementia among older adults with MCI. No association was found between single-task gait speed and progression to dementia over six years of follow-up. However, a high dual-task-walk cost while counting backward or naming animals was independently associated with a greater risk of progression to dementia.

Taken together, these findings indicate that a systematic assessment of gait patterns may serve as a powerful and easily accessible tool for the early detection of cognitive decline in clinical practice.

## 5. Conclusions

Alterations in circulating levels of several biological markers of aging have been shown to allow the identification of individuals at risk of cognitive impairment. For instance, increases in systemic levels of the inflammatory markers IL-6 and CRP are associated with MCR, a condition involving a remarkable risk of progression toward dementia. Measurement of plasma levels of NfL, a marker of neuroaxonal damage, may also assist in identifying individuals in the early stages of cognitive deterioration. Likewise, plasma concentrations of myostatin, PI3, TFF3, and PAPPA have been shown to identify at-risk individuals with high accuracy and well before clinical signs of cognitive impairment appear. Third-generation DNA methylation measures of biological aging may also be used to estimate the risk of future cognitive decline. More recently, specific circulating brain-derived EVs have been discovered that could be exploited as predictive biomarkers of neurodegeneration.

Several gait parameters can be used as powerful tools for the prediction of cognitive disorders in clinical practice. A systematic assessment of gait parameters, including a single-task walk at both a comfortable and a fast pace as well as a dual-task challenge with simultaneous walking and cognitive tasks, is an inexpensive and readily available method for the early detection of individuals at risk of cognitive impairment. The predictive value of gait measurements for cognitive impairment may be enhanced by adding measures of spatiotemporal gait characteristics such as cadence, step width, stride length, variability of stride time and length, swing time, and double-support time.

Altogether, the available evidence indicates that biological and physical performance markers may enable the identification of individuals at risk of cognitive disorders far earlier than clinical signs or symptoms become detectable. Once a condition of risk is determined, efforts should be made to enhance or maintain cognitive reserves via the elimination or control of modifiable risk factors for dementia. Future studies are needed to establish whether a combination of biological and physical performance markers allow better prediction of incident cognitive impairment than the assessment of either domain alone. Further research is also necessary to evaluate whether changes in biomarkers are sensitive to preventive interventions against dementia and may therefore be used to monitor treatment efficacy.

## Figures and Tables

**Table 1:** Original studies of biomarkers of aging related to cognitive frailty or cognitive decline.

Biomarker	Study Design and Population	Main Results	Reference
Inflammatory Markers			
CRP	Cross-sectional; individuals ≥60 years (*n* = 5642)	Higher CRP levels were associated with MCR with memory impairment.	Bai et al. (2021) [48]
Panel of inflammatory cytokines and growth factors	Cross-sectional; individuals ≥80 years without severe dementia (*n* = 415)	IL-6 was associated with cognitive and physical function.	Adriaensen et al. (2014) [49]
IL-6	Cross-sectional; individuals ≥60 years (*n* = 1340)	Individuals with cognitive frailty had significantly higher serum IL-6 levels compared with controls.	Diniz et al. (2022) [50]
CRP, IL-6, TNF-α	Cross-sectional; individuals ≥65 years without dementia (*n* = 1041)	High CRP and IL-6 serum levels were associated with MCR.	Bortone et al. (2021) [51]
	Meta-analysis of five cross-sectional studies; older adults (*n* = 3101)	Circulating IL-6 and CRP were associated with MCR, with associations varying according to the presence of vascular disease.	Groeger et al. (2022) [52]
GDF15	Cross-sectional; individuals ≥35 years (*n* = 2736)	Higher plasma GDF15 levels were associated with a combination of cognitive frailty and depression and with cognitive frailty and depressive symptoms separately in younger and older adults.	Kochlik et al. (2023) [53]
Progranulin, GDF15, IL-10, IL-6, TNF-α	Cross-sectional; prefrail adults ≥60 years without dementia (*n* = 397)	Serum TNF-α was significantly elevated in MCR independent of sarcopenia but without obesity. Low IL-10 and IL-10/TNF-α ratio were associated with MCR, independent of sarcopenia and obesity.	Merchant et al. (2023) [54]
*IL-10* gene polymorphism	Longitudinal (follow-up 3 years); individuals ≥65 years without dementia (*n* = 530)	Single-nucleotide polymorphisms in the transcriptional regulatory regions of *IL-10* gene were associated with incident MCR.	Sathyan et al. (2017) [55]
Markers of Neuroaxonal Injury			
NfL	Cross-sectional; individuals ≥45 years with SCD, MCI, or AD (*n* = 110)	Plasma NfL levels were increased in participants with MCI or AD compared with those with SCD.	Giacomucci et al. (2022) [56]
	Longitudinal (follow-up 14 years); individuals ≥55 years without dementia (*n* = 4444)	Higher plasma NfL levels were associated with greater risk of all-cause dementia or AD. Mean NfL concentrations increased 3.4 times faster in participants who developed AD compared with those who remained dementia-free. Plasma values for cases diverged from controls 9.6 years before AD diagnosis.	de Wolf et al. (2020) [57]
Extracellular Vesicles			
Total, neural-, and microglial-derived EVs	Cross-sectional; individuals with and without dementia and frailty, age not reported (*n* = 60)	Participants with AD had diminished plasma neural EVs levels. Microglial-derived EVs were increased in number in plasma of MCI participants with frailty.	Visconte et al. (2023) [58]
MicroRNAs			
Exosomal miRNAs	Longitudinal; four datasets of individuals with and without dementia (*n* = 544)	A predictive model with six miRNAs (miR29c-5p, miR-143-3p, miR-335-5p, miR-485-5p, miR-138-5p, miR-342-3p) detected preclinical AD 5 to 7 years before the onset of cognitive impairment.	Jia et al. (2022) [59]
miRNAs	Longitudinal; two datasets of individuals with and without dementia (*n* = 147)	The study found that miR-92a-3p, miR-181c-5p, and miR-210-3p were upregulated in plasma of individuals with MCI or AD compared with cognitively healthy participants. Those with MCI who progressed to AD during follow-up showed higher plasma levels of these miRNAs.	Siedlecki-Wullich et al. (2019) [60]
miRNA-206	Longitudinal (follow-up 5 years); individuals with MCI and AD (*n* = 79)	miRNA-206 was associated with cognitive decline and memory deficits. Changes in plasma levels of miRNA-206 predicted cognitive decline and progression towards dementia in participants with MCI.	Kenny et al. (2019) [61]
	Longitudinal (follow-up 5 years); individuals with amnestic MCI (*n* = 458)	During the follow-up, AD was diagnosed in 128/458 participants (28%). Serum levels of miRNA-206 were significantly higher in participants who converted to AD than in those with stable MCI both at baseline and at five years. Serum miRNA-206 was an independent predictor of AD conversion.	Xie et al. (2017) [62]
Epigenetic Clocks			
DunedinPACE	Cross-sectional; individuals ≥55 years with and without dementia (*n* = 649) Longitudinal (follow-up 14 years; *n* = 2264)	DunedinPACE was associated with clinical diagnosis of AD and worse cognitive tests. Participants with more advanced age on the clocks and faster DunedinPACE at baseline were at increased risk of developing dementia during the follow-up.	Sugden et al. (2022) [63]
DNA methylation	Longitudinal (follow-up 15 years); individuals ≥55 years with and without dementia (*n* = 52)	A lower delta age (DNAm age—chronological age) was observed in those with maintained memory functions compared with participants with average or accelerated decline. DNAm age at follow-up, but not chronologic age, was a predictor of dementia.	Degerman et al. (2017) [64]
Proteomic Markers			
Plasma proteins	Longitudinal (follow-up 15 years); individuals 20–102 years with and without dementia (*n* = 997)	Myostatin, peptidase inhibitor 3, trefoil factor 3, and pregnancy-associated plasma protein A were associated with cognitive decline in participants who were cognitively healthy at baseline.	Tanaka et al. (2020) [65]
Plasma autoantibodies	Cross-sectional; individuals ≥55 years with and without MCI (*n* = 236)	Autoantibody biomarkers differentiated participants with MCI from age- and sex-matched controls (accuracy 100%). The autoantibody panel also differentiated those with MCI from participants with mild to moderate AD or other neurologic and non-neurologic diseases.	DeMarshall et al. (2016) [66]
	Cross-sectional; individuals ≥55 years with and without MCI and dementia (*n* = 127)	Differential expression analysis identified 33 altered autoantibodies in participants with dementia compared with cognitively healthy controls, and 38 autoantibodies in those with dementia compared with individuals with MCI.	Ehtewish et al. (2023) [67]

Abbreviations: AD, Alzheimer’s disease; CRP, C-reactive protein; DNAm, DNA methylation; EVs, extracellular vesicles; GDF15, growth differentiation factor 15; IL, interleukin; MCI, mild cognitive impairment; miRNA, microRNA; MCR, motoric cognitive risk syndrome; NfL, neurofilament light chain; SCD, subjective cognitive decline; TNF-α, tumor necrosis factor alpha.

**Table 2:** Longitudinal studies on associations between frailty or gait parameters and incident cognitive decline.

Biomarker	Study Population and Follow-Up	Frailty, Cognition, and Gait Measures	Main Results	Reference
Frailty				
Physical or cognitive frailty	Cognitively healthy adults ≥65 years (*n* = 2737); follow-up 4 years	Physical frailty: handgrip strength, BMI, ASM, gait speed, chair-stand test. Cognition: MMSE.	Most frailty measures at baseline were associated with lower MMSE scores four years later.	Auyeung et al. (2011) [90]
	Cognitively healthy adults ≥65 years (*n* = 1045); follow-up 3 years	Physical frailty: Fried’s frailty criteria. Cognition: MoCA.	Chances of incident cognitive decline were more that twofold greater in individuals with physical frailty than in those with no frailty.	Chen et al. (2018) [86]
	Cognitively healthy adults ≥60 years (*n* = 385); follow-up 7 years	Frailty: Rockwood’s frailty index. Cognition: a neuropsychological test battery.	Frailty was associated with incident decline in global cognition independent of brain atrophy and cerebral small vessel disease.	Siejka et al. (2022) [87]
	Cognitively healthy adults ≥60 years (*n* = 2150); follow-up 3.5 and 7 years	Reversible cognitive frailty: presence of physical frailty and SCD. Cognition: a cognitive test battery.	Over a 3.5-year and a 7-year follow-up, participants with reversible cognitive frailty showed an increased risk of incident dementia, particularly vascular dementia.	Solfrizzi et al. (2017) [88]
	Cognitively healthy adults ≥65 years (*n* = 4570); follow-up 3 years	Physical frailty: slow gait speed and muscle weakness. Cognition: a cognitive test battery.	Cognitive frailty, but not physical frailty without MCI, was a predictor of incident dementia.	Shimada et al. (2018) [89]
Gait Measures				
Neurologic gait	Cognitively healthy adults ≥75 years (*n* = 422); follow-up 6.6 years	Gait: neurological gait assessment. Cognition: a neuropsychological test battery and clinical assessment.	The presence of neurologic gait at baseline was a predictor of dementia, especially vascular dementia.	Verghese et al. (2002) [91]
Gait speed	Cognitively healthy adults ≥70 years (*n* = 1478); follow-up 4 years	Gait: usual gait speed. Cognition: a neuropsychological test battery.	A faster gait speed at baseline was associated with less cognitive decline.	Mielke et al. (2013) [92]
	Cognitively healthy adults ≥65 years (*n* = 660); follow-up 3 years	Gait: usual and fast gait speed, walking-while-talking. Cognition: incident cognitive impairment defined as a ≥3 points loss on MMSE.	Gait speed at fast pace was associated with cognitive performance at follow-up.	Deshpande et al. (2009) [93]
	Cognitively healthy adults ≥60 years (*n* = 2654); follow-up 6 years	Gait: usual gait speed. Cognition: a cognitive test battery.	Better performance on executive function, memory, and processing speed was associated with slower decline in gait speed.	Gale et al. (2014) [94]
	Cognitively healthy adults ≥75 years (*n* = 1462); follow-up 7 years	Gait: usual gait speed. Cognition: a cognitive test battery.	Gait speed was associated with incident dementia independent of body composition parameters.	Van Kan et al. (2012) [95]
	Cognitively healthy adults ≥65 years (*n* = 1042); follow-up 2 years	Gait: usual gait speed. Cognition: 10-word delay recall test.	A slower baseline gait speed was associated with poorer cognition at follow-up.	Ojagbemi et al. (2015) [96]
	Cognitively healthy adults ≥65 years (*n* = 175); follow-up 14 years	Gait: usual gait speed. Cognition: clinical assessment.	Gait slowing was associated with cognitive impairment at year 14. A decreased gray matter volume in the right hippocampus on brain MRI was associated with both gait slowing and cognitive impairment.	Rosso et al. (2017) [97]
	Individuals ≥65 years with MMSE ≥21(*n* = 2070); follow-up 7 years	Gait: usual gait speed. Cognition: MMSE.	Participants with slower gait speed at baseline had a greater rate of cognitive decline at follow-up.	Alfaro-Acha et al. (2007) [98]
Gait speed and variability	Individuals ≥70 with and without dementia (*n* = 427); follow-up 5 years	Gait: steady state walking using an electronic system. Cognition: a neuropsychological test battery.	Higher gait variability at baseline was associated with increased risk of incident dementia.	Verghese et al. (2007) [99]
	Cognitively healthy adults ≥65 years (*n* = 758); follow-up 2 years	Gait: steady-state walking using an electronic system. Cognition: a cognitive test battery. Frailty: Fried’s frailty criteria.	Slower gait speed, lower balance confidence, and greater double-support time during walking at baseline were associated with incident cognitive frailty.	Hwang et al. (2023) [100]
	Cognitively healthy adults ≥60 years (*n* = 91); follow-up 4 years	Gait: steady-state walk using triaxial accelerometry-based gait analysis. Cognition: a standardized diagnostic interview.	Individuals with high gait variability had about a 12-fold greater risk of incident MCI than those with mid to low variability.	Byun et al. (2018) [101]
Dual-task walk	Individuals ≥70 years with MCI (*n* = 112); follow-up 6 years	Gait: steady-state single- and dual-task walking using an electronic system. Cognition: a neuropsychological test battery.	A high dual-task walk cost was associated with progression to dementia.	Montero-Odasso et al. (2017) [102]
MCR	Individuals ≥65 years with and without cognitive impairment (*n* = 314); follow-up 1–2 years	Gait: steady-state walking using an electronic system. Cognition: a neuropsychological test battery.	At baseline, MCR was associated with deficits in attention, language, and overall cognitive status. A slow gait speed and a high gait variability were associated with incident cognitive impairment.	Allali et al. (2016) [103]
	Cognitively healthy adults ≥60 years (*n* = 26,802); follow-up 12 years	Gait: steady state walking. Cognition: incident cognitive impairment defined as ≥4 points loss on MMSE.	MCR predicted incident cognitive impairment and dementia.	Verghese et al. (2014) [40]
	Cognitively healthy adults ≥60 years (*n* = 4326); follow-up 2.5 years	Gait: self-reported slow gait. Cognition: incident cases of dementia identified from insurance data.	MCR was associated with a greater risk of incident dementia.	Doi et al. (2017) [104]
	Cognitively healthy adults ≥70 years (*n* = 997); follow-up 3 years	Gait: steady-state walking using an electronic system. Cognition: a neuropsychological test battery.	Participants with MCR were at greater risk of developing dementia and vascular dementia.	Verghese et al. (2013) [45]

Abbreviations: ASM, appendicular skeletal muscle mass; BMI, body mass index; MCI, mild cognitive impairment; MCR, motoric cognitive risk syndrome; MMSE, Mini Mental State Examination; MoCA, Montreal Cognitive Assessment; MRI, magnetic resonance imaging; SCD, subjective cognitive decline.

## Data Availability

Not applicable.
